# Validation of arteriovenous access stage (AVAS) classification: a prospective, international multicentre study

**DOI:** 10.1093/ckj/sfae272

**Published:** 2024-08-30

**Authors:** Katerina Lawrie, Petr Waldauf, Peter Balaz, Ricardo Lacerda, Emma Aitken, Krzysztof Letachowicz, Mario D'Oria, Vittorio Di Maso, Pavel Stasko, Antonio Gomes, Joana Fontainhas, Matej Pekar, Alena Srdelic, VAVASC Study Group, Franchesco Ianche, Vitor Nunes, Bretislav Fabian, Jennifer Hanko, Agnes Masengu, Conor Moran, Damian McGrogan, Aidan Murray, Stephen O'Neill

**Affiliations:** Department of Transplantation Surgery, Institute for Clinical and Experimental Medicine, Prague, Czech Republic; Third Faculty of Medicine, Charles University in Prague, Prague, Czech Republic; Third Faculty of Medicine, Charles University in Prague, Prague, Czech Republic; Department of Anaesthesiology and Resuscitation, University Hospital Královské Vinohrady, Prague, Czech Republic; Third Faculty of Medicine, Charles University in Prague, Prague, Czech Republic; Division of Vascular Surgery, University Hospital Královské Vinohrady, Prague, Czech Republic; Cardiocenter, University Hospital Královské Vinohrady, Third Faculty of Medicine, Charles University, Prague, Czech Republic; Department of Vascular Surgery, National Institute for Cardiovascular Disease, Bratislava, Slovakia; RL Vascular Surgery and Interventional Radiology, Private Practice, Salvador, Brazil; Department of Renal Surgery, Queen Elizabeth University Hospital, Glasgow, UK; Department of Nephrology and Transplantation Medicine, Wroclaw Medical University, Wroclaw, Poland; Division of Vascular and Endovascular Surgery, Cardio-Thoracic-Vascular Department, University Hospital of Trieste, Trieste, Italy; Nephrology and Dialysis Unit, Department of Medicine, ASUGI – University Hospital of Trieste, Trieste, Italy; AdNa s.r.o., Vascular Surgery Clinic, Košice, Slovakia; Department of General Surgery, Hospital Professor Doutor Fernando Fonseca, Amadora, Portugal; Department of General Surgery, Hospital Professor Doutor Fernando Fonseca, Amadora, Portugal; Centre for Vascular and Mini-invasive Surgery, Hospital AGEL, Třinec-Podlesí, Czech Republic; Department of Physiology, Faculty of Medicine, Masaryk University, Brno, Czech Republic; Division of Nephrology and Haemodialysis, Internal Medicine Department, University Hospital of Split, Split, Croatia; Nephrology and Dialysis Unit, Department of Medicine, ASUGI - University Hospital of Trieste, Trieste, Italy; Department of General Surgery, Hospital Professor Doutor Fernando Fonseca, Amadora, Portugal; Centre for vascular and mini-invasive surgery, Hospital AGEL, Třinec-Podlesí, Czech Republic; Belfast City Hospital, Belfast Health and Social Care Trust, Belfast, United Kingdom; Belfast City Hospital, Belfast Health and Social Care Trust, Belfast, United Kingdom; Altnagelvin Hospital, Glenshane Road, Londonderry, United Kingdom; Belfast City Hospital, Belfast Health and Social Care Trust, Belfast, United Kingdom; Centre for Medical Education, Queen's University Belfast, Belfast, United Kingdom; Centre for Medical Education, Queen's University Belfast, Belfast, UK; Department of Transplant Surgery and Regional Nephrology Unit, Belfast City Hospital, Belfast, UK

**Keywords:** arteriovenous fistula, classification system, haemodialysis access, multicentre study, vascular mapping

## Abstract

**Background:**

The arteriovenous access stage (AVAS) classification provides evaluation of upper extremity vessels for vascular access (VA) suitability. It divides patients into classes within three main groups: suitable for native fistula (AVAS1) or prosthetic graft (AVAS2), and patients not suitable for conventional native or prosthetic VA (AVAS3). We validated this system on a prospective dataset.

**Methods:**

A prospective, international observational study (NCT04796558) involved 11 centres from 8 countries. Patient recruitment was from March 2021 to January 2024. Demographic data, risk factors, vessels parameters, VA types, AVAS class and early VA failure were collected. Percentage agreement was used to assess predictive ability of AVAS (comparison of AVAS and created VA) and consistency of AVAS assessment between evaluators. Pearson's Chi-squared test was used for comparison of early failure rate of conventional (predicted by AVAS) and unconventional (not predicted by AVAS) VA.

**Results:**

From 1034 enrolled patients, 935 had arteriovenous fistula or graft, 99 patients did not undergo VA creation due opting for alternative renal replacement therapies, experiencing health complications, death or non-compliance. AVAS1 had 91.2%, AVAS2 7.2% and AVAS3 1.6% of patients. Agreement between evaluators was 89%. The most frequently created VAs were radial-cephalic (46%) and brachial-cephalic (27%) fistulae. The accuracy of AVAS versus created access was 79%. In comparison, VA predicted by clinicians versus created access was 62.1%. Inaccuracy of AVAS prediction was more common with higher AVAS classes, and the most common reason for inaccuracy was creation of distal VA despite less favourable anatomy (17%). Patients with unconventional VA had higher early failure rate than patients with conventional VA (20% vs 9.3%, respectively, *P* = .002)

**Conclusion:**

AVAS is effective in predicting VA creation, but overall accuracy is reduced at higher AVAS classes when the complexity of decision-making increases and proximal vessels require preservation. When AVAS was followed by clinicians, early failure was significantly decreased.

## Graphical Abstract

**Graphical Abstract:**
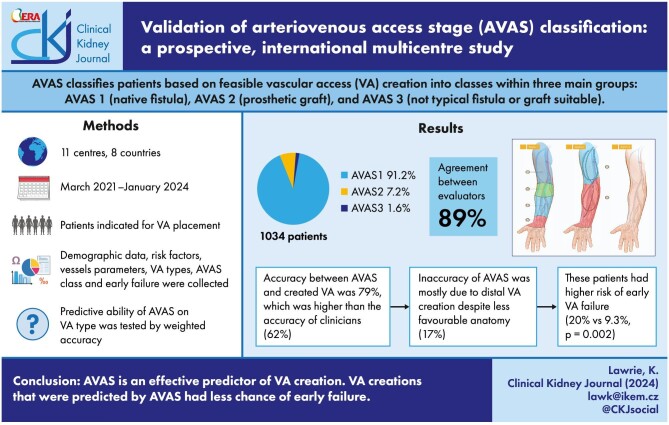

KEY LEARNING POINTS **What was known:**

- Communication within multidisciplinary teams caring for vascular access (VA) patients suffers from the lack of standardized terminology.
- Arteriovenous access stage (AVAS) classification provides information about upper extremity vessels and their suitability for VA placement.
- AVAS has been successfully applied in a retrospective analysis of patients at a single centre.

**This study adds:**

- The AVAS classification was applied in a prospective multicentre international study.
- All patients were successfully classified without exception and with a high weighted accuracy of assessment between investigators.
- AVAS proved to be a good predictor of VA creation, although with increasing AVAS class, and when surgeons spared proximal vessels the overall weighted accuracy of VA prediction was reduced.

**Potential impact:**

- The AVAS classification system can improve efficiency of communication across multidisciplinary teams, which could help to streamline care and avoid errors.
- The AVAS classification could be used to define VA populations, allowing for more accurate comparisons of healthcare unit performance and appropriate remuneration for the cost of care.
- Developing a simpler version of AVAS could make it even more user-friendly for clinical application.

## INTRODUCTION

A well-functioning vascular access (VA) is a necessity for patients who depend on haemodialysis (HD) [1]. To prevent complications, early attention to the planning process of VA is critical [2, 3]. Effective management involves timely indication, thorough vascular mapping, and preoperative examination leading to selection of the most suitable VA type for each patient [4, 5]. The aim is to establish a functional VA and an uncomplicated patient experience of dialysis [6].

**Figure 1:**
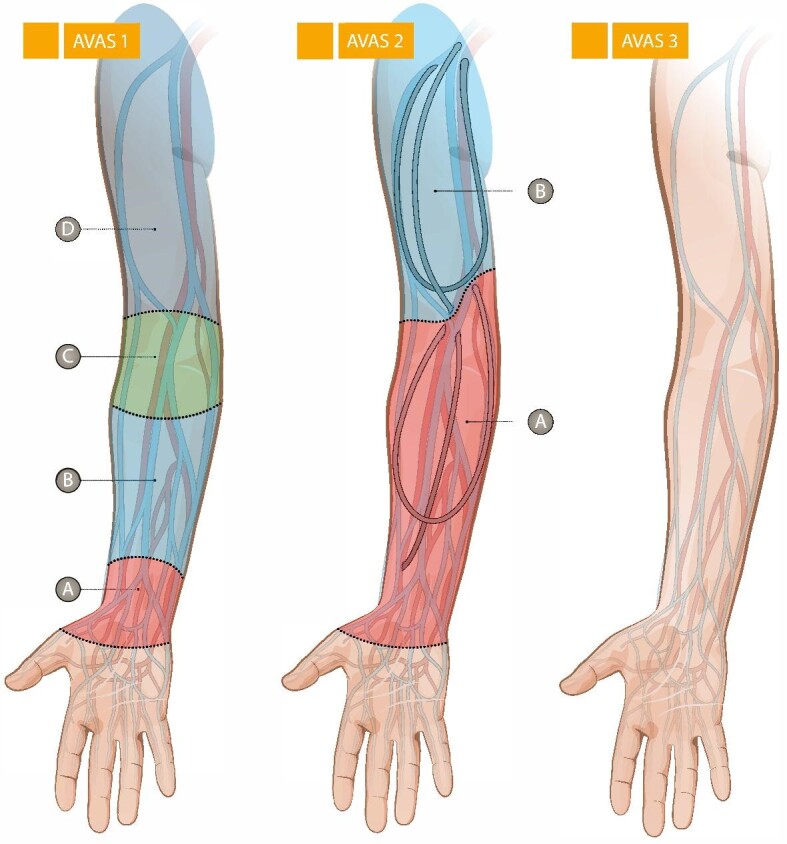
AVAS classification system, from Baláž *et al*. [8].

One of the most crucial factors in the decision-making process is the quality of veins and arteries in the upper extremity. It involves multiple factors and suffers from unstandardized communication among specialists [7]. To facilitate this communication a classification system known as arteriovenous access stage (AVAS) classification was developed [8]. This system streamlines communication of the anatomical dispositions. It aims to combine clinical assessment and duplex ultrasound vessel mapping descriptions by dividing up patients based on the feasibility of creating different types of VA. It has three main groups: patients suitable for native fistulae (AVAS1) or prosthetic grafts (AVAS2), and patients not suitable for conventional native or prosthetic VA (AVAS3). The groups AVAS1 and AVAS2 are further divided based on suitable outflow veins and their locations, which are indicated by letters A–D. The letter corresponds to the area where VA can be ideally placed (Fig. 1) [8]. This detailed classification has been successfully applied in retrospective analysis of patients at a single centre [1]. However, for its adoption in wider clinical practice, it should be validated on a prospective dataset across other centres to confirm its accuracy and clinical relevance.

The working hypothesis is that the AVAS classification system is a useful tool for sharing information about what type of VA can be created. To verify this hypothesis, we compare the AVAS classes assessed prior to surgery and the types of created VAs.

Additionally, we evaluate the accuracy of AVAS assessment between VA specialists and early failure rates of VAs in relation to AVAS.

## MATERIALS AND METHODS

### Study design

This is a prospective, observational, multicentre, international study, titled the VAVASC (Validation of Arterio Venous Access Stage Classification) study. It is registered in clinical trials registry (NCT04796558) and involves 11 centres from 8 countries (details in the Supplementary data, Table S1).

### Settings

The study has been conducted from March 2021. The recruitment of the patients was terminated on 31 January 2024. The ongoing observation of these patients is still in progress. Each centre obtained their own approvals (e.g. ethical and research governance) for data collection. The data were entered in an anonymized fashion into an electronic database (EK-VP/06/0/2021).

### Participants

All patients aged 16 years and older indicated for VA placement were eligible. The patients were enrolled consecutively. The only exclusion criterion was a patient’s refusal to have their data included in the study.

### Variables

The collected data involved demographic and patient comorbidities, ultrasonographic vascular mapping in the upper extremity and Allen's test. The details are presented in the Supplementary  data, Table S2.

### Ethical approval

The study was approved by Ethics Committee of University Hospital Královské Vinohrady in Prague, Czech Republic (EK-VP/06/0/2021).

### Outcome measures

#### AVAS class

All patients were categorized according to the AVAS classification system, both by a lead researcher at each participating centre and by the principal investigator of the VAVASC study (K. Lawrie). The criteria for AVAS assessment comply with those outlined in the original article that introduced the AVAS classification [8].

#### Type of predicted VA

Predicted VA is a type of VA recommended for the patient by VA specialist following physical examination and sonographic vascular mapping. It is not affected by AVAS as it was a decision based on the clinical judgement of the relevant clinician (e.g. when referring the patient to a surgeon for potential VA creation). The spectrum of VA types is presented in Table 1. Terminology from the Recommended standards for reports approved by the Committee on Reporting Standards of the Society for Vascular Surgery and the American Association for Vascular Surgery was used [9]. Additionally, for clinical purposes, endovascular access and proximal radial-cephalic direct access were included. Even though endovascular access has different anatomical criteria compared with surgically created VA, patients indicated for endovascular access were still consecutively recruited, evaluated and classified according to the AVAS classification system [10–12].

**Table 1:** Types of predicted and created VA.

Autogenous posterior radial branch-cephalic direct access
Autogenous radial-cephalic direct wrist access
Autogenous ulnar-basilic forearm transposition
Autogenous radial-cephalic forearm transposition
Autogenous brachial-cephalic forearm looped transposition
Autogenous radial-brachial indirect saphenous vein translocation
Prosthetic brachial-antecubital forearm loop access
Prosthetic radial-median cubital forearm straight access
Autogenous radial-cephalic direct proximal access
Autogenous brachial-cephalic upper arm direct access
Autogenous brachial-basilic upper arm transposition
Autogenous brachial-axillary indirect greater saphenous vein translocation
1st stage brachiobasilic fistula
Prosthetic brachial-axillary access
Lower extremity access procedure
Body wall access procedure
Endovascular arteriovenous fistula
None
Others

‘Direct’ refers to a native arteriovenous fistula, which is a direct connection between artery and vein. ‘Body wall access procedure’ covers tertiary arteriovenous access placed over body wall. They may be indicated in cases of central vein stenosis or occlusion that cannot be recanalized through endovascular treatment. This group involves: prosthetic axillary-axillary chest access, prosthetic axillary-axillary chest loop access, prosthetic axillary-internal jugular chest loop access, prosthetic femoral-femoral supra-inguinal access and prosthetic axillary-femoral body wall access.

#### Type of created VA

Created VA is a type of VA that was created and is also not affected by AVAS as it was a decision based on the clinical judgement of the operating surgeon. The range of VA types available for created VA is identical to the types listed for predicted VAs (Table 1).

### Bias

Efforts to diminish bias were made in the following measures. Firstly, the study was registered and the protocol was published [13]. Secondly, the data were collected prospectively by VA specialists. Lastly, the study was multicentre and involved participation from eight different countries.

### Study sample

The minimum number of patients was 800. It was deemed to be sufficient for predictive modelling and feasible to recruit within the allotted timeframe. The enrolment exceeded 1000 patients to accommodate participant dropouts from the study [13].

### Statistical methods

Continuous parameters are presented either as mean and standard deviation or as median along with the 25th and 75th percentiles, depending on whether their distribution is normal. Categorical variables are displayed as counts and percentages.

Due to the distribution of AVAS classes the predictive capability of AVAS was estimated by percentage agreement between AVAS and the type of created VA [13]. The agreement was reached if the created VA was considered conventional (based on vessel dimensions and clinical guidance) to the individual AVAS class [4, 8]. A conventional VA for a specific AVAS class is a VA that can be successfully created given the anatomical conditions with an expectation of successful maturation (where applicable) and good patency. An unconventional VA for a specific AVAS class is a VA that is not considered surgically feasible given the anatomical conditions with an expectation of non-maturation or failure if attempted.

For example, AVAS 1ABCD (‘perfect limb’) has all VA types considered as conventional due to its favourable vessels. AVAS 1CD with suitable superficial veins from the elbow proximally would be suitable for native fistulae only in the elbow and in the upper arm, but in addition prosthetic VAs, tertiary VAs and central venous lines could be considered for this class. AVAS 2B would have prosthetic VA in the arm, tertiary VAs, central venous lines considered as conventional, but not native fistulae due to insufficient superficial veins in whole limb. In total, there are 19 potential AVAS classes. The list of conventional VA types to each AVAS class is detailed in the (Supplementary data, Table S3).

A percentage agreement was also used for the accuracy of AVAS evaluation by raters. Two centres were excluded from this assessment as the principal investigator (K. Lawrie) was also evaluating VA specialist for these two centres.

To correct the uneven distribution of patients across different AVAS classes and VA types, we calculated a weighted value for every percentage agreement analysis. This involved calculating the average percentage agreement for each AVAS class and each VA type. The weighted average was calculated by taking the average of the individual averages for each AVAS category.

We tested potential differences in primary failure rates between patients whose surgeons created VA in agreement with AVAS class and those whose surgeons did not. This pertains to AVAS classes where surgeons could opt for more distal arteriovenous fistula (AVF) or arteriovenous graft (AVG) despite unsuitable anatomy, for example if a patient with AVAS 1CD (suitable elbow and upper arm AVF) had AVF created in the wrist (despite unfavourable anatomy in the wrist).

AVAS classes containing suitable distal areas (wrist and forearm) were excluded from this analysis as they do not have more distal location for comparison. The analysis involves classes with anatomically suitable areas for AVF placement in the elbow and upper arm (1C, 1CD, 1D), for prosthetic grafts (2A, 2B, 2AB) and for unsuitable conventional native or prosthetic VAs (AVAS3).

The parameter used for this evaluation was the presence of early VA failure during the first post-operative check-up. Pearson's Chi-squared test was used for comparison of early failure of conventionally and unconventionally created VAs. Statistically significant value was *P* < .05.

## RESULTS

### Patients’ characteristics

In total, 1034 patients were enrolled. The details of demographic data, clinical parameters, physical examination and mapping are presented in Table 2. Out of these patients, 935 underwent VA creation, while the other 99 patients did not receive AVF or AVG. The reasons these patients did not proceed to VA surgery are as follows: 34 patients had central venous lines inserted instead, 11 patients proceeded with peritoneal dialysis instead of haemodialysis, 10 patients managed to undergo kidney transplant before VA creation, 10 died before VA operation, 8 patients recovered or stabilized renal function eliminating their need for VA creation, 4 patients declined VA creation and 4 patients were unable to undergo VA surgery due to deterioration; 18 patients were lost to follow-up due to non-compliance.

**Table 2:** Patients’ baseline characteristics.

**Demographic data**	**Results, mean (SD) or *n* (%)**	**Unknown, *n* (%)**
Age (years)	62 (13.9)	0
Male	618 (59.8%)	0
BMI (kg/m^2^)	29 (7)	0
Weight (kg)	82.9 (20.4)	0
Clinical parameters		
Diabetes mellitus	465 (45%)	0
Smoking	225 (21.8%)	20 (1.9%)
Hypertension	767 (74.2%)	1 (0.1%)
Heart failure	147 (14.2%)	0
Ischaemic heart disease	195 (18.9%)	5 (0.5%)
History of cancer	153 (14.8%)	5 (0.5%)
Present CV line or pacemaker	362 (35%)	0
- Right side	317 (86.4%)	2 (0.5%)
- Left side	45 (12.3%)	
- Both sides	3 (0.8%)	
Previous CV line or pacemaker	203 (19.6%)	0
- Right side	132 (63.8%)	19 (9.2%)
- Left side	23 (11.1%)	
- Both sides	33 (15.9%)	
Physical examination and sonographic mapping		
Nondominant arm mapped	820 (79.3%)	42 (4.1%)
Allen's test negative	953 (92.2%)	42 (4.1%)
Arteries		
Radial artery diameter (mm)	1.92 (±1.07)	1 (0.1%)
Ulnar artery diameter (mm)	1.42 (±1.04)	3 (0.3%)
Brachial artery diameter (mm)	4.36 (±1.02)	1 (0.1%)
Patent axillary artery yes	1023 (98.9%)	0
Veins		
Cephalic vein depth in wrist and forearm area (mm)	2.48 (±2.08)	6 (0.6%)
Cephalic vein diameter in wrist and forearm (mm)	1.98 (±1.34)	0
Cephalic or basilic or median cubital vein diameter in cubital area (mm)	3.66 (±1.5)	2 (0.2%)
Basilic vein diameter in forearm (mm)	1.33 (±1.31)	3 (0.3%)
Basilic vein diameter in arm (mm)	3.13 (±1.98)	0
Patent axillary vein yes	1020 (98.6%)	0

SD, standard deviation; CV, central venous.

### Prevalence of AVAS classes

**Figure 2:**
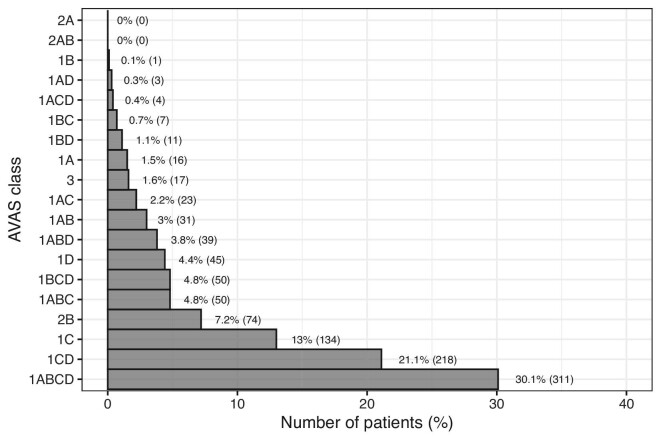
The distribution of AVAS classes.

All patients were successfully classified into one of the 19 AVAS classes. AVAS1 was assessed in 943 (91.2%), AVAS2 in 74 (7.2%) and AVAS3 in 17 (1.6%) patients. The most frequent classes were AVAS 1ABCD (whole limb suitable for all VA types) which contained 311 (30.1%) patients, 1CD (elbow and arm suitable for VA creation) with 218 (21.1%) patients and 1C (elbow area suitable for VA creation) with 134 (13%) patients. Classes 2AB and 2A (anatomically suitable for graft placement in the forearm and in whole upper extremity but not suitable for fistula) did not contain a single patient. Figure 2 illustrates the distribution details for each class of the AVAS.

### Predicted vascular access

**Figure 3:**
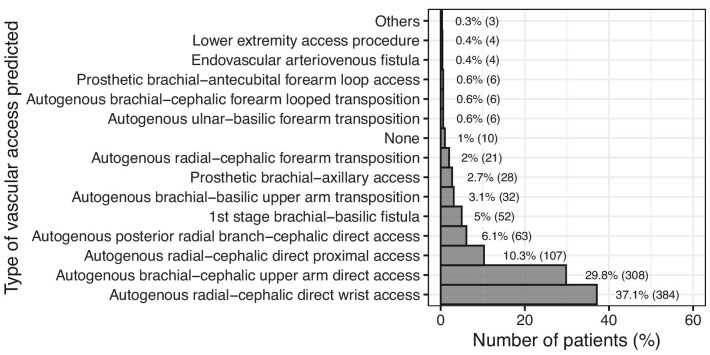
The types of predicted VA based on the clinical judgement of the relevant clinician. ‘Others’ included one autogenous radial-basilic forearm transposition (0.1%), one HeRO Graft (0.1%) and one autogenous ulnar-cephalic forearm transposition (0.1%).

The most frequent type of predicted VA was distal radial-cephalic direct wrist access (384 patients, 37.1%), followed by brachial-cephalic upper arm direct access (308 patients, 29.8%) and proximal radial-cephalic direct access (107 patients, 10.3%). A detailed diagram with all the predicted VA types is shown in Fig. 3.

### Created vascular access

**Figure 4:**
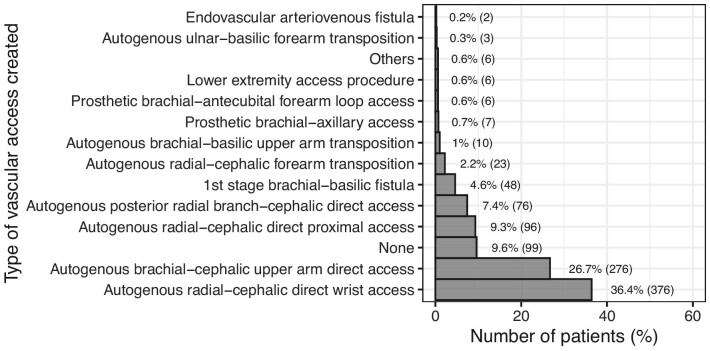
The types of created VA based on the clinical judgement of the operating surgeon. ‘Others’ included two autogenous radial-basilic forearm transpositions (0.2%), one ulnar-basilic direct access without transposition (0.1%), one ulnar-cephalic direct proximal access (0.1%), one axillary-axillary graft (0.1%) and one HeRO Graft (0.1%). ‘None’ included 11 (1.1%) central venous lines inserted while 88 patients did not have any VA placed. These patients had either another form of renal replacement therapy established, died or were lost to follow-up from our study prior to VA placement (as detailed in patient characteristics).

The most frequent created VA was distal radial-cephalic direct wrist access (376 patients, 36.4%), then brachial-cephalic upper arm direct access (276 patients, 26.7%), and then proximal radial-cephalic direct access (96 patients, 9.3%). A detailed diagram with all created VA types is in Fig. 4.

### Predictive capability of AVAS classification system

**Figure 5:**
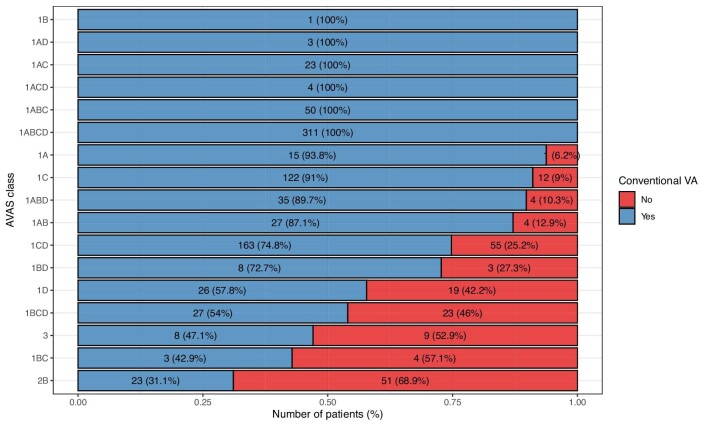
The accuracy of individual AVAS classes and created VA types expressed in weighted percentage of agreement. The X-axis displays proportion of created VA. The Y-axis portrays AVAS classes. Blue bars represent the number of VA that were conventional for the individual AVAS class. Red bars signify the number of VA that were evaluated as unconventional for each AVAS class. The total number of patients and percentage of each AVAS class are displayed within the bars.

The overall weighted accuracy between AVAS and the created VA was 78.9%. In comparison, the overall weighted accuracy of the VA predicted by clinicians and created by surgeons was 62.1% (Supplementary data, Fig. S4). The weighted accuracy of AVAS1 was 84.3%, AVAS2 31.1% (23 from 74 patients), and AVAS3 47.1% (8 from 17 patients). The highest percentage of accuracy (93.8%–100%) was found in classes containing the wrist as a suitable level for VA placement (area A): AVAS 1ABCD, 1ABC, 1ACD, 1AC, 1AD and 1A, therefore fitting mostly with radial-cephalic direct wrist access. Frequent class 1C (conventional for elbow VA) showed 91% accuracy, followed by less frequent AVAS classes containing again the wrist and forearm area (A and B) as suitable for VA creation (1ABD, 1AB) that also had high accuracy (89.7% and 87.1%, respectively). Classes that had suitability for VA placement in the upper arm (level D), 1BD, 1CD, 1BCD and 1D, had lower levels of agreement, between 54% and 74.8%. The lowest level of agreement was found in patients with AVAS 3 (not suitable for conventional AV fistula or graft), 1BC (forearm and elbow VA suitable) and AVAS 2B (conventional graft in the arm). In these groups AVAS had low accuracy of 47.1% and less. The most frequent unconventional access for patients with AVAS 1BCD (forearm and more proximal areas) and 1CD (elbow and arm areas) was radial-cephalic direct wrist access. This was found in 62 patients (62/268, 23.1%). Overall, operating surgeons opted to create a native fistula despite AVAS predicting unsuitable anatomical dispositions. The unconventional VA was opted for in 185 (185/1034, 17.9%) patients. Out of these 185 patients, more distal fistula was chosen in 88.6%, more distal graft was placed in 1.6% and native fistula or graft for challenging anatomy not conventional for typical VA (AVAS 3) was created in 4.9% of patients. In these cases, surgeons are perhaps taking a calculated risk of VA failure for other potential patient benefits (e.g. vein preservation and reduced infection). The other 4.9% of unconventional created VAs were fistulae placed more proximally than expected given the suitable anatomical conditions. Figure 5 displays specifics of percentage agreement of each AVAS class versus created VA.

### Early failure rate

An early VA failure was higher in patients, who did not have conventional VA given the AVAS class (26/130 patients, 20%). In these patients the surgeons opted for more distal VA despite less favourable anatomy. Their early VA failure rate was significantly higher than in patients with the same AVAS classes, whose surgeons placed VA in agreement with AVAS class and created conventional VA given the anatomical conditions (28/302 patients, 9.3%, *P* = .002).

### Accuracy of AVAS estimation between investigators

**Figure 6:**
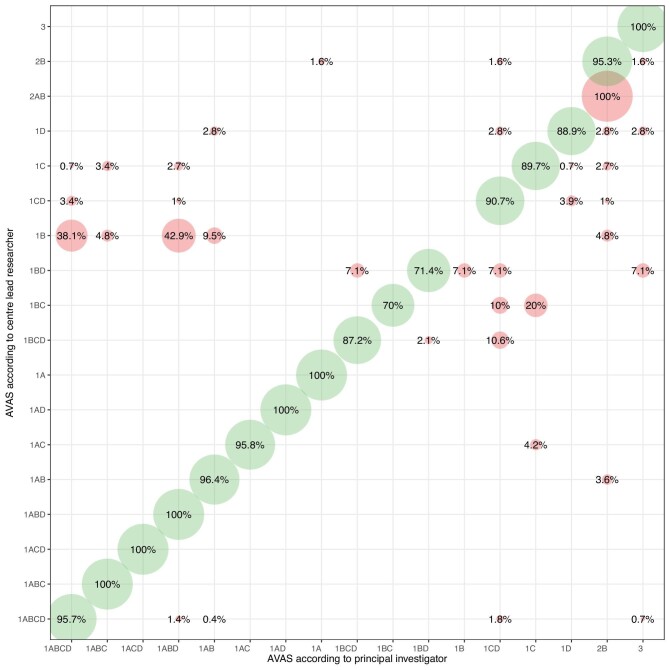
The accuracy of assessing individual AVAS classes by investigators expressed as weighted percentage of agreement. The X-axis represents the evaluation by principal investigator (K. Lawrie), while the Y-axis presents the evaluation by vascular access specialist at the centre. Green demonstrates the agreement between investigators, while red displays AVAS classes, where the evaluation of AVAS between investigators and the principal investigator differed.

The agreement level in AVAS class assessment between VA specialists and study’s principal investigator (K. Lawrie) was evaluated based on accuracy. The weighted consensus was estimated in 88.9%. The results are presented in a bubble plot, shown in Fig. 6.

## DISCUSSION

The purpose of the AVAS classification system is to provide clinicians with a straightforward method that evaluates the condition of blood vessels, thereby indicating their suitability for VA creation. It aims to improve the efficiency of communication across multidisciplinary teams, which helps to streamline care and avoid errors. The system defines VA populations, allowing for more accurate comparisons of healthcare unit performance and appropriate remuneration for the cost of care.

In clinical practice the predictive capability of AVAS in determining VA type was higher than the predictive capability of clinicians using their clinical judgment (78.9% vs 62.1%). However, AVAS has shown limitations in predictive capability potentially stemming from the personal preferences of surgeons when deciding on VA type to create. It was found that there was an increasing trend of discrepancy in prediction of created VA by AVAS with increasing AVAS class. This could imply that as the complexity of decision-making increases, the necessity for expert involvement in these decisions becomes more significant. This trend could also be explained by the fact that surgeons favour more distal native fistulae to spare the proximal ‘vascular tree’ (i.e. vein preservation) when managing more complex patients with higher AVAS class. The surgeon's approach to vein preservation is perhaps taking precedence (despite the potential increased risk of VA failure), particularly in patients with challenging anatomical conditions. A similar strategy was revealed in patients with high AVAS, e.g. 2B (graft in the upper arm suitable) and AVAS 3 (not typical cases where fistulae or grafts can be considered), when fistulas were created despite being considered not conventional type of VA by AVAS. The predictive ability of AVAS for these stages was low. This disagreement is probably affected also by the fact that the use of grafts, tertiary VAs and central venous catheters are typically associated with higher rates of complications (e.g. infection) which surgeons may be trying to avoid [1, 4, 14–17]. These findings confirm that the training and practice in VA placement are crucial. They significantly impact the decision-making process and ultimately influence VA outcomes [18, 19].

To understand how the system compares with others, we performed a systematic search of the literature and identified only three classification systems based on similar concept, each tailored to specific subgroups of VA patient populations [20]. Wilmink *et al.* (2014) introduced an elbow AVF classification, anatomically corresponding to AVAS 1C [21]. Al Shakarchi *et al.* (2015) described a system dedicated to patients with exhausted VA options, referred to as ‘end-stage VA failure’ [22]. Their system aligns with AVAS3 but provides a more detailed categorization, including assessment of central vein stenoses in both the upper and lower limbs and their specific locations [22]. Finally, Shahverdyan *et al.* (2022) presented a classification of a proximal forearm perforator for planning endovascular and surgically created Gracz type fistula, anatomically corresponding to AVAS 1BC [8, 23]. This study has shown that AVAS is broadly applicable to all patients within the HD population regardless of their anatomical conditions and displays spectrum of VAs that are technically feasible for each individual patient. In contrast, others specific classification systems focus to limited subgroups of HD populations, but they provide more detailed information about the particular VA-type placement within the specific area.

Despite the excellent recruitment, certain AVAS classes remained rare, showing that AVAS can be further simplified. This would likely further increase the agreement between clinicians when assigning AVAS from the observed level of 88.9%. A reduction in the number of classes would further establish AVAS as an easy-to-remember system for clinicians wishing to simplify communication across multidisciplinary teams. A future priority is therefore to streamline the existing AVAS system based on the distributions observed in this prospective dataset. In the next phase of our research, the aim is to also assess the outcomes and patency of VAs in relation to AVAS class, demographics and clinical factors.

### Study limitations

Even though the vessel criteria of AVAS classification adhere to recommendation of VA guidelines, we acknowledge that bias may have arisen from the vessel measurements that are on the borderline or slightly below the threshold for being considered for certain AVAS class [4]. Such cases did not achieve lower AVAS class as vessel diameters were just below the cutoff. In these instances, the clinicians may choose pragmatically to prioritize the VA option with the least risk of complications (e.g. native fistula) but also less chance of VA maturation [4, 24–28]. We also acknowledge that selection bias and confounding by indication could not be excluded due to the observational nature of the study. Lastly, the centres do not have identical VA coordination system. As a result, they had different resources, leading to varying numbers of recruited patients at each centre.

## CONCLUSION

The AVAS classification system was found to be applicable for all patients in different settings and centres. The system proved to be a good predictor for the type of VA created, although discrepancies were noted in the predictive capability with increasing AVAS class. Nonetheless, all patients were successfully categorized using the AVAS scheme, which signals its potential for clinical translation. When AVAS was followed by clinicians, early failure was significantly decreased. Future efforts will aim to develop and present a simpler version of AVAS making it even more user-friendly for clinical application, while validating the outcomes of created VAs based on their anatomical classification.

## Supplementary Material

sfae272_Supplemental_File

## Data Availability

The data underlying this article will be shared on reasonable request to the corresponding author.
