# Supplementary material for: Validation of arteriovenous access stage (AVAS) classification: a prospective, international multicentre study
Source: Clin Kidney J. 2024 Aug 30;17(9):sfae272. doi: 10.1093/ckj/sfae272 (PMC11426276; doi:10.1093/ckj/sfae272)
Supplement: sfae272_Supplemental_File [file sfae272_supplemental_file.docx]

## Appendix - Validation of Arterio Venous Access Stage (AVAS) Classification - A prospective International Multicentre Study

| **Participating centre** | **Responsible researchers** |
| --- | --- |
| Department of Transplant Surgery and Regional Nephrology Unit, Belfast City Hospital, United Kingdom | Stephen O’Neill |
| Department of Transplant Surgery, Institute for Clinical and Experimental Medicine, Prague | Katerina Lawrie |
| Division of Vascular Surgery, Cardiovascular Centre, University Hospital Královské Vinohrady, Prague, Czech Republic | Peter Balaz |
| Department of Nephrology and Transplantation Medicine, Wroclaw Medical University, Wroclaw, Poland | Krzysztof Letachowicz |
| Department of Renal Surgery, Queen Elizabeth University Hospital, Glasgow, United Kingdom | Emma Aitken |
| RL Vascular Surgery and Interventional Radiology, Private Practice, Salvador, Brazil | Ricardo Lacerda |
| AdNa s.r.o., Vascular Surgery Clinic, Košice, Slovakia | Pavel Stasko |
| Division of Vascular and Endovascular Surgery, Cardio-Thoracic-Vascular Department, University Hospital of Trieste, Trieste, Italy^1^  Nephrology and Dialysis Unit, Department of Medicine, ASUGI - University Hospital of Trieste, Trieste, Italy^2^ | Mario D’Oria^1^, Vittorio Di Maso^2^ |
| Department of General Surgery, Hospital Professor Doutor Fernando Fonseca, Amadora, Portugal | Antonio Gomes |
| Centre for Vascular and Mini-invasive Surgery, Hospital AGEL, Třinec-Podlesí, Czech Republic | Matej Pekar |
| Division of Nephrology and Haemodialysis, Internal Medicine Department, University Hospital of Split, Croatia | Alena Srdelic |

Table S1. Participating centres and responsible researchers

| Stage | Subgroup | Site | Artery* | Vein** | Allen’s test | Examples of autogenous surgical  options | 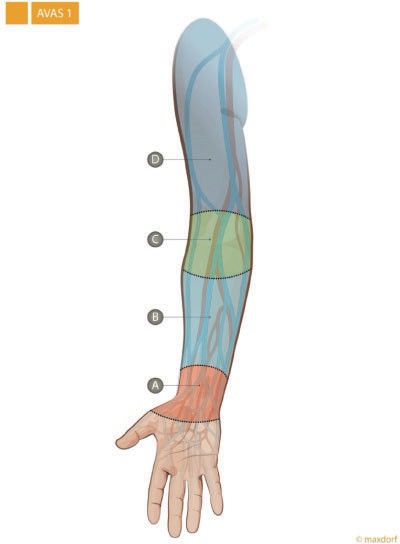 |
| --- | --- | --- | --- | --- | --- | --- | --- |
| 1  *Compressible without ≥ 50% stenosis  **Narrowing  ≤50% without phlebitis | A | Wrist | Radial ≥ 2mm | Cephalic ≥ 2mm and  ≤6mm from skin surface | Negative | Posterior radial branch- cephalic direct access (“Snuffbox ﬁstula”) |  |
|  | B | Forearm | Radial ≥ 2mm | Cephalic or basilic ≥ 2mm | Negative | Direct radial- cephalic access |  |
|  |  |  |  |  |  | Radial- cephalic forearm transposition |  |
|  |  |  |  |  |  | Radial-basilic forearm transposition |  |
|  | B | Forearm | Ulnar ≥ 2mm | Cephalic or basilic ≥  2mm | Negative | Ulnar-basilic forearm  transposition |  |
|  | C | Elbow | Brachial ≥ 3mm | Cephalic or median cubital or basilic ≥ 3mm | Not applicable | Brachial- cephalic  Brachial- basilic (1^st^ stage) |  |
|  |  |  |  |  |  | Gracz ﬁstula |  |
|  | D | Upper arm | Brachial ≥ 3mm | Basilic ≥ 3mm | Not applicable | Brachial- basilic transposition |  |

Figure S1. AVAS 1 indicates the possibility of autogenous vascular access in different parts of the upper extremity. A negative Allen’s test is demonstration of a complete palmar arch and intact collateral blood flow to the hand. From Balaz et al., (1)

| Stage | Subgroup | Site | Artery* | Vein** | Allen’s test | Examples of surgical graft options | 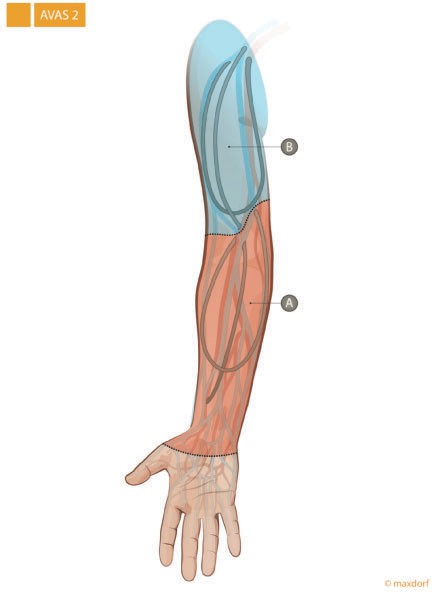 |
| --- | --- | --- | --- | --- | --- | --- | --- |
| 2  *Compressible without ≥ 50% stenosis | A | Forearm | Radial or ulnar ≥ 2mm | Antecubital  ≥ 4mm | Negative | Forearm straight |  |
|  |  | Forearm | Brachial ≥ 3mm | Antecubital  ≥ 4mm | Not applicable | Forearm loop |  |
| **Clinical and ultrasound signs of patent central veins |  |  |  |  |  |  |  |
|  | B | Upper arm | Brachial ≥ 3mm | Axillary ≥ 4mm | Not applicable | Upper arm straight |  |
|  |  | Upper arm | Axillary ≥ 3mm | Axillary ≥ 4mm | Not applicable | Upper arm loop |  |

Figure S2. AVAS 2 is reserved for patients in whom conventional autogenous vascular access is not possible and the only option is an arteriovenous graft. A negative Allen’s test is demonstration of a complete palmar arch and intact collateral blood flow to the hand. From Balaz et al.,(1).

| Stage | Subgroup | Site | Artery | Vein | Allen’s test | Examples of  surgical options | 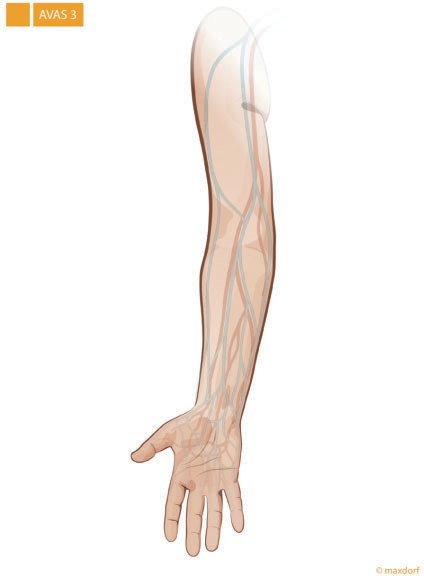 |
| --- | --- | --- | --- | --- | --- | --- | --- |
| 3 | Not applicable | Not applicable | Stenosis ≥ 50%  Non- compressible “egg shell wall” | Untreatable central vein stenosis or occlusion  Exhausted or insuﬃcient peripheral veins  Phlebitis | Not applicable | HERO graft  Arterio- arterial AVA  Brachial vein transposition |  |

Figure S3. AVAS 3 is reserved for patients without the possibility for conventionally created autogenous or prosthetic options for access creation due to insufficient venous, arterial system or its combination. A negative Allen’s test is demonstration of a complete palmar arch and intact collateral blood flow to the hand. From Balaz et al., (1).

| **Demographic data** | **Clinical parameters** |
| --- | --- |
| Age | Diabetes mellitus |
| Sex (male / female) | Smoking |
| Height [cm] | Hypertension |
| Weight [kg] | Heart failure |
| BMI | Ischaemic heart disease |
|  | Cancer |
|  | Present and previous history of CV line or pacemaker and side |
| **Physical examination and sonographic mapping** | |
| Side of the examining arm | |
| Dominant hand | |
| Allen’s test | |
| **Arteries** [mm] | **Veins** [mm] |
| Radial artery diameter | Cephalic vein depth (wrist and forearm area) |
| Ulnar artery diameter | Cephalic vein diameter (wrist and forearm area) |
| Brachial artery diameter | Cephalic or basilic or median cubital vein diameter in cubital area |
| Axillary artery patent (yes / no) | Basilic vein diameter in forearm |
|  | Basilic vein diameter in arm |
|  | Axillary vein patent (yes / no) |

Table S2. Collected parameters

| 1ABCD | Autogenous posterior radial branch-cephalic direct access  Autogenous radial-cephalic direct wrist access  Endovascular arteriovenous fistula  Autogenous ulnar-basilic forearm transposition  Autogenous radial-cephalic direct proximal access  Autogenous radial-cephalic forearm transposition  Autogenous brachial-cephalic forearm looped transposition Autogenous brachial-cephalic upper arm direct access  Autogenous brachial-basilic upper arm transposition  1st stage brachiobasilic fistula  Prosthetic brachial-antecubital forearm loop access Prosthetic radial-median cubital forearm straight access Prosthetic brachial-axillary access  Autogenous radial-brachial indirect saphenous vein translocation  Autogenous brachial-axillary indirect greater saphenous vein translocation Lower extremity access procedure Body wall access procedure Others None |
| --- | --- |
| 1ABC | Autogenous posterior radial branch-cephalic direct access  Autogenous radial-cephalic direct wrist access  Endovascular arteriovenous fistula Autogenous ulnar-basilic forearm transposition  Autogenous radial-cephalic direct proximal access  Autogenous radial-cephalic forearm transposition  Autogenous brachial-cephalic forearm looped transposition Autogenous brachial-cephalic upper arm direct access  Prosthetic brachial-antecubital forearm loop access Prosthetic radial-median cubital forearm straight access Prosthetic brachial-axillary access  Autogenous radial-brachial indirect saphenous vein translocation  Autogenous brachial-axillary indirect greater saphenous vein translocation Lower extremity access procedure Body wall access procedure Others None |
| 1ACD | Autogenous posterior radial branch-cephalic direct access  Autogenous radial-cephalic direct wrist access  Autogenous radial-cephalic direct proximal access  Autogenous brachial-cephalic upper arm direct access  Autogenous brachial-basilic upper arm transposition  1st stage brachiobasilic fistula  Prosthetic brachial-antecubital forearm loop access Prosthetic radial-median cubital forearm straight access Prosthetic brachial-axillary access  Autogenous radial-brachial indirect saphenous vein translocation  Autogenous brachial-axillary indirect greater saphenous vein translocation Lower extremity access procedure Body wall access procedure Others None |
| 1ABD | Autogenous posterior radial branch-cephalic direct access  Autogenous radial-cephalic direct wrist access  Autogenous ulnar-basilic forearm transposition  Autogenous radial-cephalic direct proximal access  Autogenous radial-cephalic forearm transposition  Autogenous brachial-basilic upper arm transposition  1st stage brachiobasilic fistula  Prosthetic brachial-antecubital forearm loop access Prosthetic radial-median cubital forearm straight access Prosthetic brachial-axillary access  Autogenous radial-brachial indirect saphenous vein translocation  Autogenous brachial-axillary indirect greater saphenous vein translocation Lower extremity access procedure Body wall access procedure Others None |
| 1AB | Autogenous posterior radial branch-cephalic direct access  Autogenous radial-cephalic direct wrist access  Autogenous ulnar-basilic forearm transposition  Autogenous radial-cephalic direct proximal access  Autogenous radial-cephalic forearm transposition  Prosthetic brachial-antecubital forearm loop access Prosthetic radial-median cubital forearm straight access Prosthetic brachial-axillary access  Autogenous radial-brachial indirect saphenous vein translocation  Autogenous brachial-axillary indirect greater saphenous vein translocation Lower extremity access procedure Body wall access procedure Others None |
| 1AC | Autogenous posterior radial branch-cephalic direct access  Autogenous radial-cephalic direct wrist access  Autogenous radial-cephalic direct proximal access  Autogenous brachial-cephalic upper arm direct access  Prosthetic brachial-antecubital forearm loop access Prosthetic radial-median cubital forearm straight access Prosthetic brachial-axillary access  Autogenous radial-brachial indirect saphenous vein translocation  Autogenous brachial-axillary indirect greater saphenous vein translocation Lower extremity access procedure Body wall access procedure Others None |
| 1AD | Autogenous posterior radial branch-cephalic direct access  Autogenous radial-cephalic direct wrist access  Autogenous brachial-basilic upper arm transposition  1st stage brachiobasilic fistula  Prosthetic brachial-antecubital forearm loop access Prosthetic radial-median cubital forearm straight access Prosthetic brachial-axillary access  Autogenous radial-brachial indirect saphenous vein translocation  Autogenous brachial-axillary indirect greater saphenous vein translocation Lower extremity access procedure Body wall access procedure Others None |
| 1A | Autogenous posterior radial branch-cephalic direct access Autogenous radial-cephalic direct wrist access |
| 1BCD | Endovascular arteriovenous fistula  Autogenous ulnar-basilic forearm transposition  Autogenous radial-cephalic direct proximal access  Autogenous radial-cephalic forearm transposition  Autogenous brachial-cephalic forearm looped transposition Autogenous brachial-cephalic upper arm direct access  Autogenous brachial-basilic upper arm transposition  1st stage brachiobasilic fistula  Prosthetic brachial-antecubital forearm loop access Prosthetic radial-median cubital forearm straight access Prosthetic brachial-axillary access  Autogenous radial-brachial indirect saphenous vein translocation  Autogenous brachial-axillary indirect greater saphenous vein translocation Lower extremity access procedure Body wall access procedure Others None |
| 1BC | Endovascular arteriovenous fistula  Autogenous ulnar-basilic forearm transposition  Autogenous radial-cephalic direct proximal access  Autogenous radial-cephalic forearm transposition  Autogenous brachial-cephalic forearm looped transposition Autogenous brachial-cephalic upper arm direct access  Prosthetic brachial-antecubital forearm loop access Prosthetic radial-median cubital forearm straight access Prosthetic brachial-axillary access  Autogenous radial-brachial indirect saphenous vein translocation  Autogenous brachial-axillary indirect greater saphenous vein translocation Lower extremity access procedure Body wall access procedure Others None |
| 1BD | Autogenous ulnar-basilic forearm transposition  Autogenous radial-cephalic direct proximal access  Autogenous radial-cephalic forearm transposition  Autogenous brachial-basilic upper arm transposition  1st stage brachiobasilic fistula  Prosthetic brachial-antecubital forearm loop access Prosthetic radial-median cubital forearm straight access Prosthetic brachial-axillary access  Autogenous radial-brachial indirect saphenous vein translocation  Autogenous brachial-axillary indirect greater saphenous vein translocation Lower extremity access procedure Body wall access procedure Others None |
| 1B | Autogenous ulnar-basilic forearm transposition  Autogenous radial-cephalic direct proximal access  Autogenous radial-cephalic forearm transposition  Prosthetic brachial-antecubital forearm loop access Prosthetic radial-median cubital forearm straight access Prosthetic brachial-axillary access  Autogenous radial-brachial indirect saphenous vein translocation  Autogenous brachial-axillary indirect greater saphenous vein translocation Lower extremity access procedure Body wall access procedure Others None |
| 1CD | Endovascular arteriovenous fistula  Autogenous radial-cephalic direct proximal access  Autogenous brachial-cephalic upper arm direct access  Autogenous brachial-basilic upper arm transposition  1st stage brachiobasilic fistula  Prosthetic brachial-antecubital forearm loop access Prosthetic radial-median cubital forearm straight access Prosthetic brachial-axillary access  Autogenous radial-brachial indirect saphenous vein translocation  Autogenous brachial-axillary indirect greater saphenous vein translocation Lower extremity access procedure Body wall access procedure Others None |
| 1C | Endovascular arteriovenous fistula  Autogenous radial-cephalic direct proximal access  Autogenous brachial-cephalic upper arm direct access  Prosthetic brachial-antecubital forearm loop access Prosthetic radial-median cubital forearm straight access Prosthetic brachial-axillary access  Autogenous radial-brachial indirect saphenous vein translocation  Autogenous brachial-axillary indirect greater saphenous vein translocation Lower extremity access procedure Body wall access procedure Others None |
| 1D | Autogenous brachial-basilic upper arm transposition  1st stage brachiobasilic fistula  Prosthetic brachial-antecubital forearm loop access Prosthetic radial-median cubital forearm straight access Prosthetic brachial-axillary access  Autogenous radial-brachial indirect saphenous vein translocation  Autogenous brachial-axillary indirect greater saphenous vein translocation Lower extremity access procedure Body wall access procedure Others None |
| 2AB | Prosthetic brachial-antecubital forearm loop access  Prosthetic radial-median cubital forearm straight access  Prosthetic brachial-axillary access  Autogenous radial-brachial indirect saphenous vein translocation  Autogenous brachial-axillary indirect greater saphenous vein translocation Lower extremity access procedure Body wall access procedure Others None |
| 2A | Prosthetic brachial-antecubital forearm loop access  Prosthetic radial-median cubital forearm straight access  Autogenous radial-brachial indirect saphenous vein translocation  Autogenous brachial-axillary indirect greater saphenous vein translocation Lower extremity access procedure Body wall access procedure Others None |
| 2B | Prosthetic brachial-axillary access  Autogenous radial-brachial indirect saphenous vein translocation  Autogenous brachial-axillary indirect greater saphenous vein translocation Lower extremity access procedure Body wall access procedure Others None |
| 3 | Lower extremity access procedure Body wall access procedure Others None |

Table S3. Individual AVAS classes and groups of conventional vascular accesses


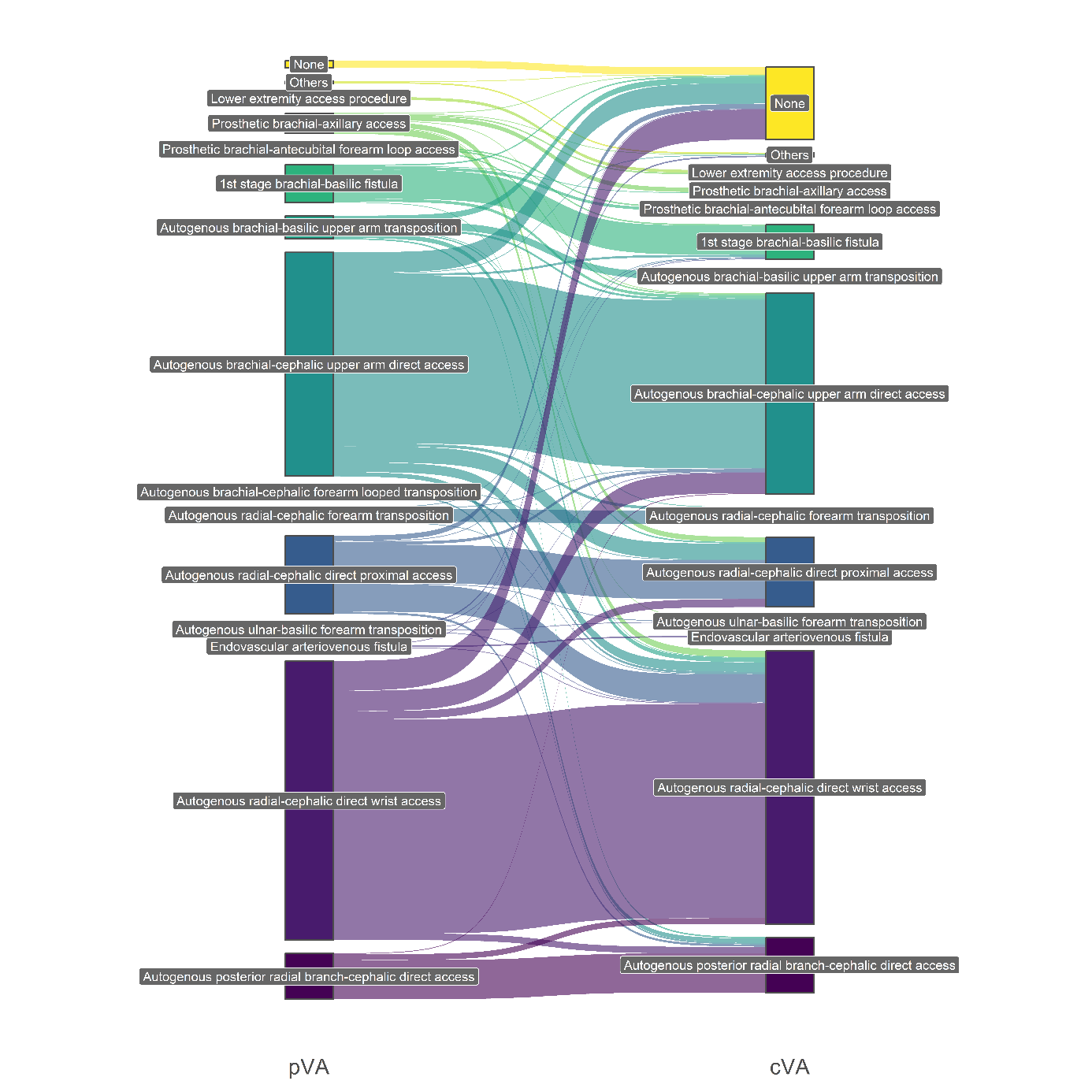


Figure S4. Sankey plot illustrating the relations between predicted and created vascular accesses. pVA = predicted vascular access, cVA = created vascular access. The weighted accuracy was 62.1 %.

## References:

1. Baláž P, Hanko J, Magowan H, Masengu A, Lawrie K, O’Neill S. The arteriovenous access stage (AVAS) classification. Clinical Kidney Journal 2021; 14: 1747–1751.
